# Differential impact of landscape‐scale strategies for crop cultivar deployment on disease dynamics, resistance durability and long‐term evolutionary control

**DOI:** 10.1111/eva.12570

**Published:** 2017-11-30

**Authors:** Julien Papaïx, Loup Rimbaud, Jeremy J. Burdon, Jiasui Zhan, Peter H. Thrall

**Affiliations:** ^1^ BioSP, INRA Avignon France; ^2^ CSIRO Agriculture & Food Canberra ACT Australia; ^3^ Fujian Key Laboratory of Plant Virology Institute of Plant Virology Fujian Agriculture and Forestry University Fuzhou China

**Keywords:** cropping ratio, durability, epidemiological control, landscape, resistance deployment strategy, spatial aggregation, spatially explicit model, susceptible‐exposed‐infectious‐removed

## Abstract

A multitude of resistance deployment strategies have been proposed to tackle the evolutionary potential of pathogens to overcome plant resistance. In particular, many landscape‐based strategies rely on the deployment of resistant and susceptible cultivars in an agricultural landscape as a mosaic. However, the design of such strategies is not easy as strategies targeting epidemiological or evolutionary outcomes may not be the same. Using a stochastic spatially explicit model, we studied the impact of landscape organization (as defined by the proportion of fields cultivated with a resistant cultivar and their spatial aggregation) and key pathogen life‐history traits on three measures of disease control. Our results show that short‐term epidemiological dynamics are optimized when landscapes are planted with a high proportion of the resistant cultivar in low aggregation. Importantly, the exact opposite situation is optimal for resistance durability. Finally, well‐mixed landscapes (balanced proportions with low aggregation) are optimal for long‐term evolutionary equilibrium (defined here as the level of long‐term pathogen adaptation). This work offers a perspective on the potential for contrasting effects of landscape organization on different goals of disease management and highlights the role of pathogen life history.

## 1 INTRODUCTION

The use of resistant crop cultivars against pathogens typically reduces epidemic development and associated yield losses in agricultural production systems. However, such cultivars may rapidly become ineffective owing to the well‐documented ability of pathogens to evolve and overcome plant resistance genes (Burdon, Zhan, Barrett, Papaïx, & Thrall, 2016; Zhan, Thrall, & Burdon, 2014; Zhan, Thrall, Papaïx, Xie, & Burdon, 2015). The development of new crop cultivars carrying novel resistance genes is usually long, expensive and frequently constrained by available resistance sources. As a consequence, there is significant merit in considering how different spatiotemporal deployment strategies for resistant cultivars may impact on resistance durability (Burdon, Barrett, Rebetzke, & Thrall, 2014; Gilligan & van den Bosch, 2008). To maximize any advantages to be gained, such approaches should explicitly address and integrate epidemiological and evolutionary perspectives of pathogens (Thrall et al., 2011), noting that the definition of an optimal strategy may vary between different stakeholders for crop production systems. The epidemiological perspective is focused on reductions in pathogen population size and the severity of disease, and consequently its impact on farm profitability. In this context, the goal is for resistance deployment to persist as long as possible; that is, resistances must be still effective after a long period of use, despite the pathogen's evolutionary abilities. However, the medium to long‐term control and the short‐term epidemiological control offered by a resistant cultivar are not necessarily correlated (Johnson, 1984). Therefore, the design of deployment strategies that optimize both epidemiological and evolutionary outcomes is not an easy task, and both criteria must be used to fully assess the performance of different disease management options (Burdon et al., 2014; Zhan et al., 2015).

The epidemiological impact of using a resistant plant cultivar on pathogen populations mostly depends on the type of resistance. Two resistance types are usually described in the literature. Qualitative resistance, also called “major‐gene resistance,” refers to the gene‐for‐gene model (Flor, 1955). This resistance relies on the noncompatible interaction of a specific host resistance protein with a pathogen avirulence effector, usually leading to complete immunity of the host (i.e., avirulent strains of the pathogen cannot successfully infect resistant hosts, while they can infect susceptible hosts). When deployed homogeneously across large areas and over extended time periods, qualitative resistance imposes strong and directional selection on pathogen populations. In such situations, new infectivity may quickly emerge in pathogen populations, resulting in total breakdown of the resistance gene (Johnson, 1983, 1984). In contrast, quantitative resistance results from the additive effect of multiple minor genes. It does not block infection but constrains disease as compared to susceptible cultivars (Mundt, 2014) due to reduced pathogen fitness (e.g., colonization, reproduction and transmission—Brown, 2015; Lannou, 2012; Parlevliet, 2002; Stuthman, Leonard, & Miller‐Garvin, 2007). In this system, the loss of resistance efficiency is best described as a progressive erosion, due to pathogen evolution consisting of an increase in one or more aggressiveness traits (Brown, 2015; Mundt, 2014). In either qualitative or quantitative host–pathogen interactions, evolution of a pathogen towards increased infectivity or aggressiveness on a resistant host is often penalized by a fitness cost on susceptible hosts (Leach, Cruz, Bai, & Leung, 2001; Montarry, Cartier, Jacquemond, Palloix, & Moury, 2012; Thrall & Burdon, 2003). As a consequence, generalist pathogens (able to infect a wide range of host genotypes) are often less adapted to a particular host than specialist pathogens, indicating the existence of life‐history trade‐offs in many pathogens (Laine & Barrès, 2013).

As resistance breakdown or high levels of erosion results in resistant cultivars becoming ineffective, several approaches have been proposed to improve their durability including the use of chemicals, agronomic practices, varying crop diversity either temporally (i.e., crop rotations) or spatially (e.g., pyramiding, mixtures, mosaics of fields), or combinations of some or all of these cultural practices (Brown, 2015; Burdon et al., 2014; Mundt, 2014; Stuthman et al., 2007; Zhan et al., 2015). The efficiency of some of these approaches has been experimentally evaluated, as exemplified by the large‐scale assessment of rice mixtures to control rice blast in China (Zhu et al., 2000). However, empirical data are often difficult to obtain for landscape‐scale strategies in spite of considerable evidence from modelling studies that landscape organization can affect pathogen population size and impede adaptation to host resistance (for reviews, see Plantegenest, Le May, & Fabre, 2007; Real & Biek, 2007). Empirical evidence of the impact of landscape organization originates mostly from studies in natural systems (Allan, Keesing, & Ostfeld, 2003; Condeso & Meentemeyer, 2007; Haas, Hooten, Rizzo, & Meentemeyer, 2011; Langlois, Fahrig, Merriam, & Artsob, 2001). For example, these studies have shown that spatiotemporal heterogeneity in environmental conditions (including host genetic structure) plays a crucial role in determining the potential for species and genotypes to coexist and in shaping the evolution of populations and species. Host–pathogen systems are no exception; metapopulation processes were shown to be key determinants of observed patterns of disease and genetic diversity (Burdon & Thrall, 1999; Jousimo et al., 2014; Laine, Burdon, Dodds, & Thrall, 2011; Smith, Ericson, & Burdon, 2003; Soubeyrand, Laine, Hanski, & Penttinen, 2009; Tack & Laine, 2014).

In the few simulation models developed to investigate the case of agricultural systems, most describe agricultural landscapes of susceptible and resistant cultivars allocated to different fields as a mosaic. In this way, the impact of several landscape characteristics (e.g., cultivar composition within fields, aggregation of fields sharing the same composition, presence of a wild reservoir) on disease control was evaluated in different pathosystems. One general result that emerges from many spatially explicit but purely demographic models (Holt & Chancellor, 1999; Papaïx, Touzeau, Monod, & Lannou, 2014; Papaïx, Adamczyk‐Chauvat et al., 2014; Skelsey, Rossing, Kessel, & van der Werf, 2010) is that an “optimal” landscape in the context of epidemiological control of disease should be composed of a high proportion of fields occupied by the resistant cultivar, hereafter referred to as the “cropping ratio” (i.e., the proportion of fields where the resistant cultivar is deployed) and a low degree of landscape aggregation (i.e., the mean proportion of neighbouring fields that share the same cultivar). The effect of aggregation is, however, not straightforward as highly aggregated landscapes can perform better when considering quantitative resistances (Papaïx, Touzeau et al., 2014).

In models that explicitly integrate pathogen evolution, the impact of cropping ratio may strongly depend on the type of host resistance (Lo Iacono, van den Bosh, & Paveley, 2012) and epidemic intensity (Fabre, Rousseau, Mailleret, & Moury, 2012, 2015). However, as these models (Fabre et al., 2012, 2015; Lo Iacono et al., 2012) are not spatially explicit, the effect of landscape aggregation could not be explicitly evaluated. In addition, Fabre et al. (2015) did not simultaneously evaluate epidemiological and evolutionary measures of the effectiveness of different strategies. However, van den Bosch and Gilligan (2003) emphasized that different measures of resistance durability (time to establishment of a population carrying a new infectivity, infectivity frequency and yield) could potentially be affected in different ways by the cropping ratio of the resistant cultivar. Using a demogenetic model to study how the spatial and temporal distribution of remnant wild vegetation patches embedded in an agricultural landscape can influence the ability of a pathogen to evolve onto a crop, Papaïx, Burdon, Zhan, and Thrall (2015) also found that the emergence of a crop pathogen and its subsequent specialization on the crop host were impacted in a different way by landscape organization (composition and spatial structure of remnant wild vegetation patches). Indeed, landscape organizations that promoted larger pathogen populations on the wild host facilitated the emergence of a crop pathogen, but such landscape organizations also reduced the potential for the pathogen population to adapt to the crop.

Here, we developed a spatially explicit model to study the impact of landscape organization on both the efficiency and evolutionary durability of crop resistance following deployment. Although the model was loosely based on rusts, a group of foliar fungal diseases consisting many economically important pathogens (Chen, Wellings, Chen, Kang, & Liu, 2014; Park, 2008), our aim was to examine how spatial heterogeneity of resistance may shape the epidemiology and evolution of agricultural pathogens in general contexts rather than focusing on any particular pathosystem. We thus provide here a theoretical analysis of an ideal situation where the landscape structure is simple and remains unchanged across time to focus on the role of spatial heterogeneity in shaping the epidemiology and evolution of agricultural pathogens. Using this approach, a resistant cultivar consisting of a pyramid of both qualitative and quantitative resistances, and a susceptible cultivar are allocated to different fields as a mosaic across an agricultural landscape. The choice of considering a resistant cultivar carrying both qualitative and quantitative resistances was motivated by empirical evidences of increased durability of qualitative resistance when combined with quantitative ones (e.g., Brun et al., 2010). Three model outputs are used as optimization criteria for this resistance deployment strategy: short‐term epidemiological dynamics (defined here as the average proportion of healthy plants for the susceptible cultivar before the resistant cultivar loses its immunity), resistance durability (defined here as the first year the resistant variety loses its immunity) and long‐term evolutionary equilibrium (defined here as the stable level of long‐term pathogen adaptation). The model is stochastic and based on the Susceptible‐Exposed‐Infectious‐Removed (SEIR) architecture to describe the life cycle of the pathogen. In addition to the impact of landscape organization, we also assessed the influence of some life‐history traits of the pathogens on the different outputs.

## 2 MODEL AND STATISTICAL ANALYSES

### 2.1 Model

#### 2.1.1 Model overview and definitions of disease risk

The model we developed describes the epidemiological and evolutionary dynamics of a pathogen population in an agricultural landscape. It assumes that the pathogen disperses passively across the whole landscape (e.g., via wind dispersed propagules). The landscape is composed of fields where a susceptible crop cultivar and a resistant crop cultivar are sown in controlled proportions and degree of spatial aggregation. The crop is present all year‐round although plant cover is reduced during the off‐season. For a given simulation, the spatial structure of the landscape is assumed to remain the same across years.

The two crop cultivars impose selection pressure on the pathogen population through a single life‐history trait, the efficacy of infection. Thus, pathogen genotypes are characterized by their associated ability to infect the two host cultivars. We assumed that the resistant cultivar consists of a pyramid of both qualitative and quantitative resistances. Thus, at the beginning of a simulation, the pathogen population is only adapted to the susceptible cultivar and cannot attack the resistant cultivar. However, new pathogen genotypes can progressively arise through mutation, resulting in the emergence of genotypes associated with gradually increasing infection efficacy on the resistant cultivar. These new pathogen genotypes are only partially adapted to the resistant cultivar because of the presence of quantitative resistance. In addition, they are penalized by decreasing infection efficacy on the susceptible cultivar to account for life‐history trade‐offs, as has been documented for several rust pathogens (Laine & Barrès, 2013).

**Figure 1:**
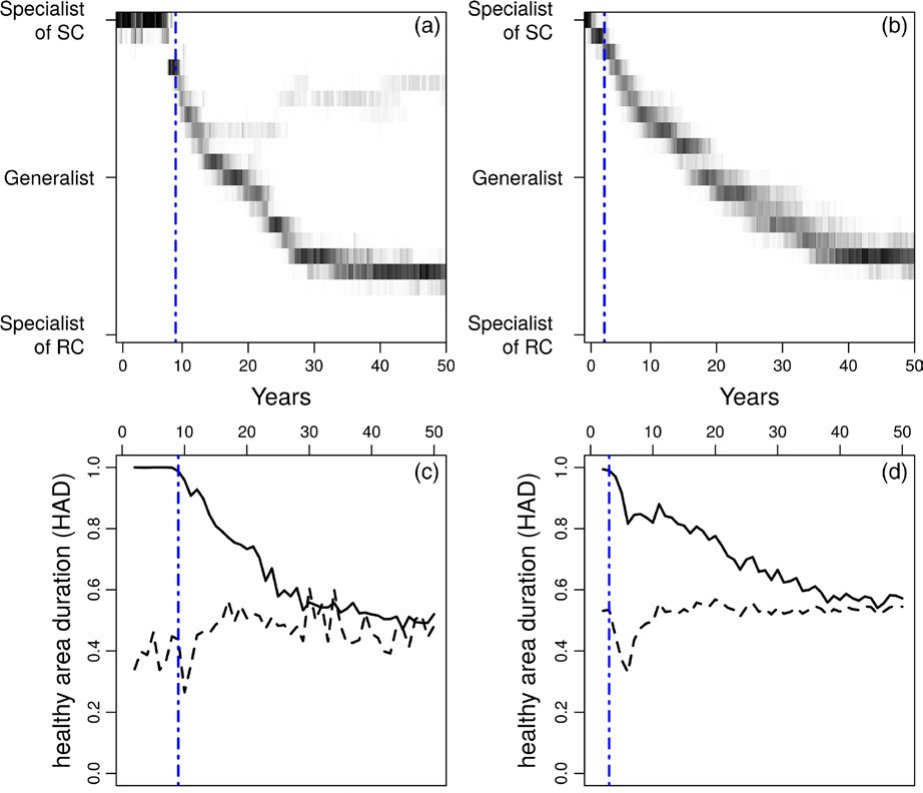
Genetic composition (frequencies of the different genotypes, black = 100%, white = 0%) of the pathogen population (a and b, RC = resistant cultivar, SC = susceptible cultivar); and evolution of the healthy area duration (HAD, c and d, 1 = no disease, 0 = maximum of disease) during 50 years of simulation for the susceptible cultivar (dashed line) and the initially resistant cultivar (solid line). The blue line indicates the time when the resistant cultivar loses its immunity (referred as resistance durability). The values of the parameters used in these simulations are as follows: β = 1, *r *=* *5 and $\mu_{0}=10$ (a, b, c and d); the landscape is composed by 90% of the resistant cultivar with a grouped aggregation (a and c), or by 70% of the resistant cultivar with a mixed aggregation (b and d)

Using this modelling framework in a theoretical context, we quantified the impacts of the cropping ratio of the resistant cultivar and its spatial aggregation on three measures representing different phases of pathogen adaptation to the resistant cultivar (Figure 1): short‐term epidemiological dynamics, resistance durability and long‐term evolutionary equilibrium. For this, the healthy area duration (HAD)—the integral of healthy green canopy area during the yield forming period (Waggoner & Berger, 1987)—of plant cover, for either the susceptible cultivar or the resistant cultivar, is computed by integrating, each year, the proportion of healthy individuals during the cropping season. This reflects the cumulative photosynthetic tissue available for grain production and filling. Then, the three model outputs are computed after a 50‐year simulation (see Figure 1 for an example) as follows. The short‐term epidemiological dynamics is assessed by computing HAD for the susceptible cultivar and averaged over the period from the beginning of the simulation to the time when the resistant cultivar loses its immunity (following the emergence of pathogen genotypes able to infect the resistant cultivar). In this study, this time is defined as the first year when HAD of the resistant cultivar dropped by 5% and is thereafter referred as resistance durability. Finally, the long‐term evolutionary equilibrium is assessed at the end of the simulation by averaging HAD of both cultivars over the final 5 years, assuming that pathogen evolutionary dynamics reached their equilibria.

#### 2.1.2 Landscape model

**Figure 2:**
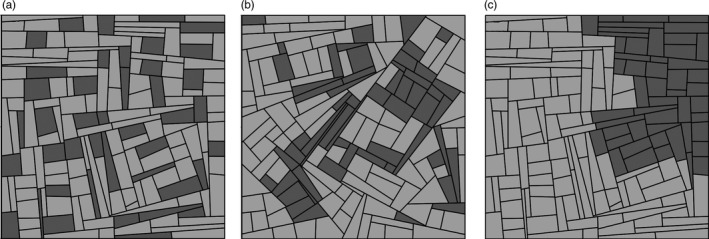
Simulated landscapes with 30% of the crop being represented by the resistant cultivar and an increasing aggregation level (a: low; b: intermediate; c: high)

A landscape pattern composed of approximately 155 fields is generated by simulating a set of fields using a T‐tessellation algorithm that makes it possible to control the size, number and shape of fields (Kiêu, Adamczyk‐Chauvat, Monod, & Stoica, 2013; Papaïx, Adamczyk‐Chauvat et al., 2014). Following this step, a susceptible crop cultivar and a resistant crop cultivar are deployed across the simulated landscape using controlled spatial arrangements defined by their proportions in terms of surface coverage (10%, 30%, 50%, 70% and 90% of the resistant cultivar) and aggregation level (Figure 2). The landscape patterns are replicated five times, and the allocations of cultivars to fields are replicated twice, by means of a simulated annealing algorithm. Although simulated landscapes do not represent the full complexity of agricultural systems, their use in theoretical studies makes it possible to consider a variety of landscapes with controlled features as well as stochastic variations in the landscape structure rather than a unique situation, which limits the generality of the results (Papaïx, Adamczyk‐Chauvat et al., 2014).

#### 2.1.3 Pathogen demogenetic dynamics

We consider a Susceptible‐Exposed‐Infectious‐Removed (SEIR) model with foliar sites as individuals (i.e., a site where a lesion can develop). The host population is composed of two genotypes (corresponding to the susceptible and resistant cultivars) and the pathogen population of *p* genotypes. The initial pathogen genotype corresponds to the full specialist on the susceptible cultivar (it cannot infect the resistant cultivar) and the other genotypes correspond to those associated with gradually increasing infection efficacy on the resistant cultivar. These genotypes are considered as generalists with different degrees of specialization as they can infect both cultivars, at least to some extent. The model then describes the dynamics of the number of foliar sites in each of the following states and for each field *i* (*i *=* *1,…,*I*): healthy sites (*S*_*i*_), latent sites infected by pathogens of genotype *p* (*E*_*i,p*_), infectious sites infected by pathogens of genotype *p* (*I*_*i,p*_) and removed sites infected by pathogens of genotype *p* (*R*_*i,p*_). Spores produced by infectious sites correspond to the propagule state.

Epidemics were simulated over 50 years, each composed of 12 months of 30 days. This duration was chosen to ensure detection of the long‐term impact of the deployment strategy on disease epidemics and pathogen evolution. The cropping season formed the first 120 days of the year, whereas the off‐season was represented by the remaining 240 days of the year. Below, we describe each sequential step of the model as it occurs during a cropping season. The full description is given in the Appendix S1.

##### Initial conditions

For each simulation, the pathogen population is initially composed of the specialist of the susceptible cultivar (*p *=* *1). Epidemics are initiated by assuming that plants in susceptible fields are randomly infected with a probability of .01 (infected plants at *t *=* *0 are at an infectious stage).

##### Reproduction and mutation

Infectious sites produce *r *=* *5 or 10 effective spores per day resulting from the number of spores effectively landing on new hosts after dispersal, and the propensity of hosts to be infected (Soubeyrand, Sache, Lannou, & Chadoeuf, 2007). Spores are associated with the same genotype as their parental lesion with probability *m*_pp_ = 0.996. When a mutation occurs (with *p *=* *.004), we assume that the pathogen population evolves gradually: a new genotype arises from closely related genotypes by mutation with small gains or losses in infection efficacy by setting *m*_*p*(*p*‐1)_ = *m*_*p*(*p*+1)_ = 0.002. The exceptions are pathogen genotypes with the highest infection efficacy on either susceptible or the resistant cultivars—these mutate towards less specialized genotypes with a probability of .004 to ensure their overall mutation rate is equal to that of other genotypes.

##### Spore dispersal

Spores migrate from field *i* to field *j* with probability μ_*ij*_ computed from:

$$\mu_{i,j}=\frac{\int_{A_{i}}\int_{A_{j}}g\left(||z-z^{\prime }\left|\right|\right)dzdz^{\prime }}{A_{i}},$$

Where *A*_*i*_ and *A*_*j*_ are the areas of fields *i* and *j*, respectively (Bouvier, Kiêu, Adamczyk, & Monod, 2009). $g\left(||z-z^{\prime }\left|\right|\right)$ is the individual dispersal function with an inverse power law shape:

$$g\left(||z-z^{\prime }\left|\right|\right)=\frac{\left(a-2)(a-1\right)}{2\pi b^{2}}{\left(1+\frac{||z-z^{\prime }\left|\right|}{b}\right)}^{-a},$$

where $||z-z^{\prime }\left|\right|$ is the Euclidean distance between locations *z* and z′ *b *>* *0 is a scale parameter and *a *>* *2 determines the weight of the dispersal tail. The parameter *a* is fixed at 3.4 to simulate a fat‐tailed dispersal function. The mean dispersal distance is defined by μ=2b/(a−3) and is varied to represent 2.5%, 10% and 25% of the landscape length (noting that the simulated landscape is square).

##### Invasion of healthy sites

Spores arriving in a field invade a healthy site with probability $\pi (x_{i}\left(t\right))$, where π(·) is an increasing function of *x*_*i*_(*t*), the proportion of healthy sites in field *i* at time *t*. Indeed, all of the infection sites on an individual plant are not equally accessible to spores, for instance because of the plant physical structure. Then, the possible new infections are distributed among the pathogen genotypes according to their proportion in the set of spores arriving in the field *i* at time *t* and following a multinomial distribution.

##### Infection of invaded sites

A healthy site receiving a spore (invaded site) becomes infected with probability *e*_*p,v(i)*_ the infection efficacy associated with pathogen genotype *p* on crop cultivar *v*(*i*) cultivated in field *i*.

We assume a trade‐off in infection efficacy on the two crop cultivars (respectively, *e*_*p*,RC_ and *e*_*p*,SC_ for the resistant and the susceptible cultivars): a gain in infection efficacy on the resistant cultivar has a cost in terms of reduced infection efficacy on the susceptible cultivar (and vice versa). Gain and cost are linked through the relationship (Débarre & Gandon, 2010):

(1) $$e_{p,\mathrm{RC}}=e_{\max}-1+{\left(1-{e_{p,\mathrm{SC}}}^{\frac{1}{\beta }}\right)}^{\beta }$$

with *e*_max_ = 0.4 the infection efficacy of a fully specialist and β the global shape of the trade‐off curve: the curve is concave when β is below unity, linear when β = 1 and convex otherwise. We will refer hereafter to concave curves as weak trade‐offs, because they correspond to cases where the cost of being a generalist is low. Similarly, convex curves will be referred to as strong trade‐offs; β can then be referred as the trade‐off strength (Ravigné, Dieckmann, & Olivieri, 2009). The infection efficacies associated with the different pathogen genotypes are computed from Equation (1), by varying the infection efficacy on the resistant cultivar between 0 and *e*_max_ and by considering three values for the trade‐off strength, β = 0.8, β = 1 and β = 1.2.

##### Transition from latent (E) to infectious (I) sites

Once infected, the invaded sites remain latent for an average of τ=5 days (Azzimonti, 2012) before becoming infectious.

##### Removal of infectious sites

After an average of *T *=* *10 days of sporulation (i.e., the length of the infectious period; Azzimonti, 2012), infectious sites are removed and unable to produce new propagules.

##### Host growth and removal of sites

To initiate a cropping season, plant cover is set to 10% of the field acreage. The crop then grows locally until it reaches the carrying capacity of the field, *K*_*i*_, where *K*_*i*_ is assumed to be proportional to the area of field *i*. In the following, results are expressed as ratios so that they are independent of the constant of proportionality (except for when very low carrying capacities are considered, in which case demographic stochasticity can be very important, which is not the case in this study). In addition, we consider that only healthy sites (*S*_*i*_) contribute to biomass production (equivalent to a castrating pathogen). At the end of the cropping season an arbitrary proportion of 99.9% of the sites is removed randomly, regardless of their infection state, and the total number of sites is kept constant during the off‐season (i.e.*,* no host growth).

### 2.2 Statistical analyses

Simulations were performed using a complete factorial design to explore the five input parameters of interest: cropping ratio (five values—10%, 30%, 50%, 70% and 90%), landscape aggregation (three values—low, intermediate and high), strength of the trade‐off between infection efficacies (three values—0.8, 1 and 1.2), spore production rate (two values—5 and 10 spores.day^−1^) and mean distance of spore dispersal (three values—2.5%, 10% and 25% of the landscape length). For each combination of these five parameters, 20 replicates were simulated (five landscape pattern replicates, two allocation replicates and two model replicates to account for stochasticity). This resulted in a total of 5400 simulations.

**Figure 3:**
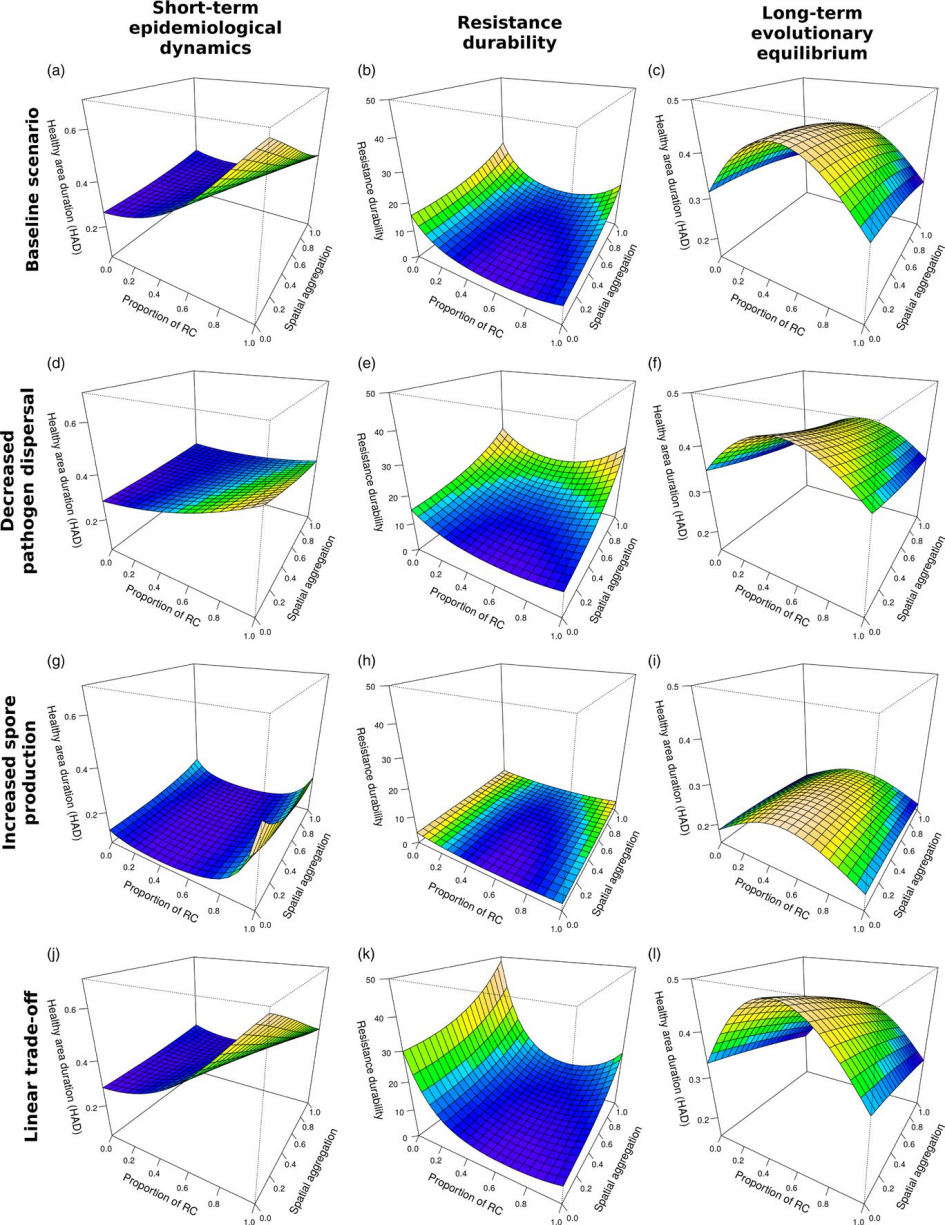
Relationship between landscape organization (proportion of the resistant cultivar and spatial aggregation) and the three model outputs based on the computation of the healthy area duration (HAD—a, d, g and j, short‐term epidemiological dynamics; b, e, h and k, resistance durability; c, f, i and l, long‐term evolutionary equilibrium). A baseline scenario (a, b and c—values of parameters: β = 0.8, *r *=* *5 and $\mu_{0}=25\%$) is compared to a scenario with decreased pathogen dispersal (d, e and f—values of parameters: β = 0.8, *r *=* *5 and $\mu_{0}=2.5\%$), with increased spore production (g, h and i—values of parameters: β = 0.8, *r *=* *10 and $\mu_{0}=25\%$) and with a linear trade‐off (j, k and l—values of parameters: β = 1, *r *=* *5 and $\mu_{0}=25\%$)

Firstly, we analysed the simulations by fitting generalized linear models to the three model outputs including the effect of the five input parameters and their second order interactions. Stepwise selection based on the Bayesian information criterion (BIC) allowed us to retain the most parsimonious models (Table 1). Secondly, we computed the total sensitivity indices of the different input parameters, relative to the three model outputs (Table 1). Lastly, we put emphasis on the role of landscape organization (cropping ratio and landscape aggregation) using meta‐models consisting of second degree Legendre polynomials (Sudret, 2008). This allowed us to establish the response surfaces (Figure 3).

**Table 1:**
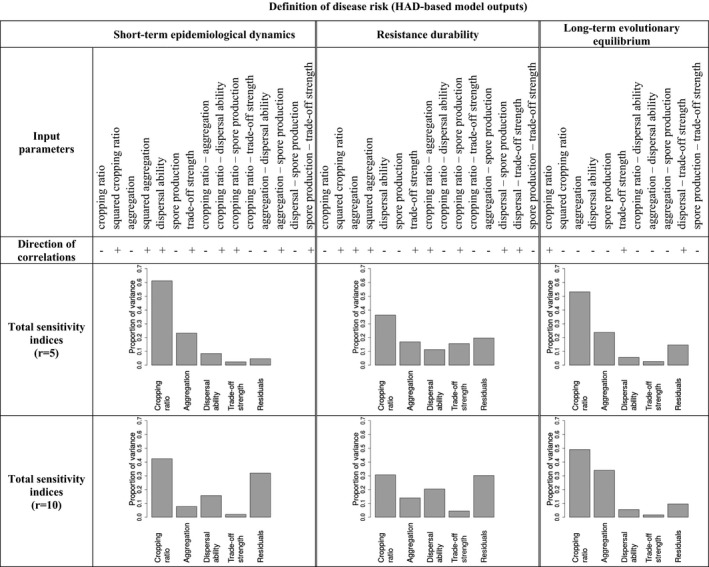
Best models retained after the stepwise selection based on the Bayesian information criterion (BIC) for the three model outputs computed from the healthy area duration (HAD), along with the direction of correlations between input parameters and model outputs and effect sizes (total sensitivity indices)

## 3 RESULTS

In all, 7.2% of simulations were discarded because the pathogen population went extinct in the initial stages of the simulation as a result of demographic stochasticity during the off‐season (when only a few hosts were available). Pathogen extinction occurred most frequently in mixed landscapes with a high proportion of the resistant cultivar and when life‐history trait parameters were clearly to the disadvantage of the pathogen (low dispersal ability, low spore production and a strong trade‐off).

In the remaining simulations, the epidemics are characterized by an initial period of immunity of the resistant cultivar (Figure 1). Then, new infectivity emerges in the pathogen population through mutation. From this point time, the qualitative component of resistance is overcome and the quantitative component begins to erode through subsequent mutations in the pathogen genotypes able to overcome qualitative resistance (Figure 1).

### 3.1 Short‐term epidemiological dynamics

In the short term (during the phase when the resistant crop cultivar is still immune to disease), the HAD of the susceptible cultivar ranged from 11.7% to 73.2% over all simulations (the median was ~50%). From the GLM analysis, a lower amount of disease in the short term occurred with lower spore production capacity (Table 1). In addition, higher dispersal ability was associated with lower disease in the short term (Table 1) probably due to an increased alloinfection/autoinfection ratio. The trade‐off strength was not retained in the best model (Table 1). The adjusted response surfaces indicated that both cropping ratio and the spatial aggregation of crop cultivars have a strong impact on short‐term epidemiological dynamics (Figure 3 and Table 1). As expected, we found that the HAD of the susceptible cultivar increases with greater proportion of the resistant cultivar and lower spatial aggregation. The cropping ratio had the largest effect with the proportion of the immune cultivar explaining 60% (*r *=* *5) or 40% (*r *=* *10) of the variability in short‐term disease dynamics (Table 1).

### 3.2 Resistance durability

The emergence of infectivity in the pathogen population was observed in 88.5% of the simulations. In almost 40% of the simulations, the resistant cultivar lost its immunity after only 1 or 2 years (the median was 5 years) postintroduction. Increases in pathogen dispersal ability and spore production decreased resistance durability while stronger trade‐offs resulted in more durable resistance (Table 1). Resistance durability was the highest with low cropping ratios (i.e., deployment of the resistant cultivar on a low proportion of fields; Figure 3 and Table 1). Interestingly, we found a positive effect of spatial aggregation (Figure 3 and Table 1): increases in the aggregation of fields where the resistant cultivar was sown increased the durability of resistance. The sensitivity analysis showed that the cropping ratio was always the most influential parameter on resistance durability (Table 1).

### 3.3 Long‐term evolutionary equilibrium

Following the emergence of new infectivity in pathogen populations, both cultivars were susceptible to disease (at least to some extent) and the HAD of the agricultural landscape at the end of the simulation was dependent on the global level of adaptation of the pathogen population. Over all simulations, the HAD of the entire landscape ranged from 17.2% to 62.4% (the median was 26.7%). In 72.6% of simulations, two pathogen genotypes coexisted in the population at the end of the simulation; each of them being more adapted to one of the two cultivars (e.g., Figure 1a). In the other 27.4% of simulations, only one pathogen genotype was maintained in the population (e.g., Figure 1b). Consistent with the results for resistance durability, increases in either pathogen dispersal ability or spore production capacity increased the long‐term level of adaptation of the pathogen while stronger trade‐offs led to reduced levels of adaptation (Table 1). The long‐term evolutionary equilibrium leading to the lower amount of disease was achieved by combining both cultivars in balanced proportions with low spatial aggregation (Figure 3 and Table 1). Depending on the values of the pathogen life‐history traits, such landscape organization may result in either the selection of one generalist pathogen or the selection of two different genotypes but with a low degree of specialization. The sensitivity analysis showed that the cropping ratio was always the most influential parameter but aggregation still explained between 25% (*r *=* *5) and 35% (*r *=* *10) of the variability of long‐term evolutionary equilibrium (Table 1).

## 4 DISCUSSION

To go beyond the blanket deployment of resistance genes, the sustainable management of crop resistance to disease needs a better understanding of the demogenetic dynamics of pathogen populations from the initial release of a new resistant cultivar to the breakdown/erosion of its resistance and further adaptation of pathogen populations. The present study is a first attempt to characterize, using a theoretical framework, the dynamics of HAD following the deployment of an initially resistant cultivar and a susceptible cultivar across a landscape (with a deployment strategy combining pyramiding and mosaic as well as qualitative and quantitative resistance) in response to pathogen demography and evolutionary dynamics. This study is based on a stochastic and spatially explicit SEIR model applied to a foliar fungal disease as typified by cereal rusts. It offers a perspective on the strong differences that may exist between management strategies of resistance deployment accounting for different measures of disease risk, derived either from epidemiological or evolutionary perspectives.

The effect of the combination of susceptible and resistant cultivars at field (mixtures) and landscape (mosaic) scales on disease development has been classically studied in the literature (Mundt, 2002; Papaïx, Touzeau et al., 2014; Skelsey et al., 2010). It has generally been found that the best epidemiological control is obtained when the cropping landscape is composed of a high proportion of the resistant cultivar and weak spatial aggregation. However, these results are typical of mixtures and mosaics governed by qualitative plant–pathogen interactions but must be mitigated when considering quantitative plant–pathogen interactions as spillover from the susceptible to the partially resistant variety may decrease disease control in landscapes with low spatial aggregation (Papaïx, Touzeau et al., 2014). Our results are in agreement with these studies as low spatial aggregation was found to increase HAD. Note that our short‐term criterion deals only with complete resistance as it was calculated over the period when the resistant cultivar was still immune to disease.

The optimal spatial structure of the landscape is different when our second criterion (resistance durability) is taken into account. Durability of resistance genes as a function of the deployment strategy has previously been studied using nonspatial models (but see Sapoukhina, Tyutyunov, Sache, & Arditi, 2010 for an example at the field scale). The basic finding of such studies is that a low cropping ratio of the resistant cultivar minimizes selection pressure on the pathogen population and thus increases the durability of the resistance gene (Fabre et al., 2012; Pietrevalle, Lemarié, & van den Bosch, 2006; Pink & Puddephat, 1999). However, high cropping ratios can also delay the breakdown of resistance by drastically reducing pathogen population size, resulting in a U‐shaped function of durability against cropping ratio (van den Bosch & Gilligan, 2003). Using a spatially explicit model, we also found such a pattern but with low cropping ratios always being more durable. However, our model has refined this result on the effects of cropping ratio as increasing spatial aggregation and thus decreasing fragmentation of the landscape were found to favour resistance durability. Indeed, in our simulations epidemics typically proceeded on the resistant cultivar via spillover from the susceptible cultivar. Thus, more aggregated spatial patterns of cultivar deployment lead to a smaller interface between susceptible and resistant crop cultivars, which limits immigration and the emergence of resistance‐breaking genotypes in the pathogen population. As a consequence of the effect of this interface, we observed a strong interaction between the cropping ratio and spatial aggregation. Indeed, the influence of cropping ratio was stronger for high levels of aggregation.

Time to emergence of new infectivity in crops treated by fungicides was studied by Bourget, Chaumont, and Sapoukhina (2013) using a nonspatial model. They identified a particular situation for which a strategy based on a small proportion of treated fields was not as durable as one based on treating a high proportion of fields. This arose if the pathogen population had a low growth rate and high migration abilities. In our model, we also found that increasing pathogen dispersal ability decreases resistance durability. However, low cropping ratios were never found to significantly decrease resistance durability compared to high cropping ratios. Indeed, in most cases, resistance durability was preserved by hindering pathogen dispersal between the resistant and susceptible crop (i.e., when dispersal was low, aggregation was high) and preferentially when the resistant crop was at low proportion. The main difference between Bourget et al. (2013) and our results comes from the fact that we did not explore situations with low pathogen growth rates as we focused on foliar fungi that typically have high growth rates. Finally, durability was found to be sensitive to the shape of the trade‐off function, which influences the ability of the pathogen to perform well on both cultivars. This is consistent with Fabre et al. (2012) who found that the cost of virulence was the most important parameter with regard to explaining resistance durability.

The long‐term evolutionary equilibrium as assessed by HAD at the end of a simulation was also found to be highly sensitive to the spatial structure of the landscape. As for qualitative resistance, increase in the area covered by the resistant cultivar is expected to increase the speed of pathogen evolution and thus the erosion of quantitative resistance (Lo Iacono et al., 2012). However, as demonstrated by Lo Iacono et al. (2012), the integrated gain on yield over time, which comes from cultivating high ratios of the resistant cultivar, may outweigh the more rapid evolution of the pathogen. In addition to the speed of resistance erosion, cropping ratio also affects the level of adaptation of the pathogen population at equilibrium. Consistent with previous studies (e.g., Papaïx, David, Lannou, & Monod, 2013), we found that balanced proportions of susceptible and resistant cultivars and low spatial aggregation reduced the long‐term impact of the disease. In the present work, we focused on the role of host spatial heterogeneity and considered that quantitative resistance only affected one pathogen trait, infection efficacy. According to Lo Iacono et al. (2012), quantitative resistance that targets infection efficacy is indeed more efficient in controlling disease (in an epidemiological sense) than quantitative resistance targeting pathogen reproduction rate (but see Bourget, Chaumont, Durel, & Sapoukhina, 2015). The role of quantitative resistance in driving pathogen adaptation is gaining attention in the literature with emphasis on determining which pathogen life‐history traits are affected by host resistance (Azzimonti, Lannou, Sache, & Goyeau, 2013; Pariaud, Ravigné et al., 2009). When a high penalty exists for the new infectivity against a resistance, compensation via other traits may operate to improve pathogen fitness on resistant hosts. In this case, susceptible cultivars may also be affected as they will suffer the effects of higher aggressiveness from the pathogen population (van den Berg, Lannou, Gilligan, & van den Bosch, 2013; Gandon, Mackinnon, Nee, & Read, 2001). The evolutionary consequences of possible correlations between life‐history traits (trade‐off, pleiotropy) are also important as they determine the adaptive landscape for pathogen populations (Lannou, 2012; Pariaud, Robert, Goyeau, & Lannou, 2009). For example, Bourget et al. (2015) showed that resistances affecting pathogen life‐history traits that are in conflict with each other are more durable.

The significance of the current study is that it jointly analyses evolutionary and epidemiological outputs of a landscape‐scale strategy of resistance deployment using a stochastic and spatially explicit model. It clearly shows that the management of disease risk through spatial organization of the agricultural landscape faces strong constraints depending on whether short‐term epidemiological dynamics, resistance durability or long‐term evolutionary equilibrium is targeted. Indeed, we found that low aggregation combined with a high proportion of the resistant cultivar is optimal for short‐term epidemiological dynamics, whereas the exact opposite (high aggregation and low cropping ratio) is optimal for resistance durability. Finally, well‐mixed landscapes (low aggregation with balanced proportions) are optimal for long‐term evolutionary equilibrium of pathogen populations.

Nevertheless, these conclusions must be nuanced by the theoretical context of this study. The target of this work was to specifically explore the short‐ to long‐term impacts of spatial heterogeneity on resistance durability and disease control. Thus, we considered that the landscape organization remained the same for the whole simulation run. In practice, the same resistance genes may be introgressed into many commercial cultivars and continuously used for long periods of time although particular cultivars containing these resistance genes may turn over frequently (Johnson, 1984; Niks, Qi, & Marcel, 2015; Stuthman et al., 2007). However, when genes are used in successive cultivars, they are generally associated with different combinations of qualitative resistances or with different genetic backgrounds, modifying the selective pressure over the period during which the genes are used (e.g., Goyeau & Lannou, 2011). More specifically, our results could be modified in different ways by the consideration of temporal heterogeneity, mainly depending on its shape (arm race vs. boom‐and‐bust cycles) and on the life‐history traits that are impacted (Débarre & Gandon, 2011). Temporal heterogeneity implies a stronger bottleneck because of the need for the pathogen to reach the new host sown to different fields in the following season, potentially leading to the loss of adaptive mutations through drift and thus delaying resistance breakdown. However, temporal heterogeneity can also increase pathogen diversity by increasing the possibilities for specialist and generalist genotypes to coexist in different geographic locations in the landscape because of asynchrony in crop rotations (Papaïx, Burdon, Lannou, & Thrall, 2014). It is then unclear if this diversity increases or not disease severity and pathogen abilities to adapt to new cultivars. In the case of directional gradual changes (e.g., due to selection of cultivars more and more resistant), the outputs are extremely sensitive to the speed at which growers are able to produce new cultivars with respect to the speed at which the pathogen evolves (Polechová, Barton, & Marion, 2009). Thus, similar to coevolving systems (Burdon & Thrall, 2009), the dynamics of resistance evolution depend also on the adaptive response to pathogen evolution of stakeholders for crop production systems (Zhan et al., 2015). Currently, crop rotations are recommended to provide disease breaks (Bennett, Bending, Chandler, Hilton, & Mills, 2012) as they may reduce pathogen population size from year to year, given that only a portion of pathogen population may successfully land and survive on newly sown susceptible fields. For example, Fabre et al. (2015) found, with a spatially implicit model for virus epidemics, that a combination of mosaics and rotations performed better than mosaics alone from both epidemiological and evolutionary perspectives. However, the efficiency of crop rotations could be limited in regions where the crop is cultivated over large areas, particularly for diseases spread by aerial primary inoculum. For example, a combination of a 6‐year rotation (to reduce the soil inoculum) with a delayed sowing (to avoid the peak of the aerial inoculum) was recommended by McDonald and Peck (2009) to control Ascochyta blight on field peas (*Pisum sativum L*.). In our model, temporal heterogeneity was considered through seasonality in host density. Indeed, for biotrophic pathogens, host harvest can represent a severe bottleneck, potentially eliminating rare adaptive mutations. We assumed that, during the off‐season, host genotypes remain the same as during the cropping season and that individual plants are homogenously distributed within a field. These assumptions limit the effect of spatial structure on both off‐season eco‐evolutionary dynamics (Tack & Laine, 2014) and the inoculum for the new cropping season (Mundt, Leonard, Thal, & Fulton, 1986) and imply homogeneity in the direction of selection between the off‐season and the cropping season (Papaïx et al., 2015). Finally, we assumed that infectivity was absent from the initial pathogen population. However, resistance genes can originate from wild relatives of crops. In that case, a pathogen population may have already been exposed to the resistance gene and consequently evolved towards new infectivity (Leroy, Le Cam, & Lemaire, 2014). The pre‐existence of infectivity in the pathogen population, even at low frequency, can change dramatically the durability of the resistance gene in the crop as well as which deployment strategy is optimal (Lof, de Vallavieille‐Pope, & van der Werf, 2017).

Future investigations using our modelling approach may include consideration of additional temporal heterogeneity in the landscape as well as the simulation of more complex resistance deployment strategies, which are likely to be better approaches to deploy plant resistance (Fabre et al., 2015). Furthermore, the use of quantitative resistance targeting different pathogen life‐history traits possibly in combination with a diversity of major resistance genes should be investigated in the context of spatiotemporal deployment strategies given its potential to keep pathogen population maladapted (Brown, 2015; Zhan et al., 2015). In addition, disease control is better achieved using a combination of agricultural practices (Meynard, Doré, & Lucas, 2002). In particular considering pesticide treatments in combination with high diversity in genetic resistance will provide additional guidance for more realistic management strategies. Lastly, the definition of management strategies for a specific agricultural region has to be based on actual landscape patterns with crop species and varieties allocated through decision rules integrating technical and socio‐economic constrains (Rounsevell & Arneth, 2011).
