# Supplementary material for: Differential impact of landscape‐scale strategies for crop cultivar deployment on disease dynamics, resistance durability and long‐term evolutionary control
Source: Evol Appl. 2017 Nov 30;11(5):705–17. doi: 10.1111/eva.12570 (PMC5979631; doi:10.1111/eva.12570)
Supplement: Supplementary file 1 [file EVA-11-705-s001.docx]

**Supplementary Material S1 – Model description**

**Differential impact of landscape-scale strategies for crop cultivar deployment on disease dynamics, resistance durability and long-term evolutionary control**

We consider a Susceptible-Exposed-Infectious-Removed (SEIR) model with foliar sites as individuals (*i.e.* a site where a lesion can develop). The host population is composed of two genotypes (corresponding to the susceptible and resistant cultivars) and the pathogen population of $P$ genotypes. The first genotype corresponds to the full specialist of the susceptible cultivar (it cannot infect the resistant cultivar) and the other genotypes correspond to genotypes associated with gradually increasing infection efficacy on the resistant cultivar. The other genotypes are considered as generalists with different degrees of specialisation since they can infect both cultivars, at least to some extent. The model then describes the dynamics of the number of foliar sites in each of the following states and for each field $i$ (*i* = 1,…,*I*): healthy sites ($S_{i}$), latent sites infected by pathogens of genotype $p$ ($E_{i,p}$), infectious sites infected by pathogens of genotype$p$ ($I_{i,p}$), and removed sites infected by pathogens of genotype$p$ ($R_{i,p}$). Spores produced by infectious sites correspond to the propagule state.

Epidemics were simulated over 50 years composed of 12 months of 30 days. The cropping season formed the first 120 days of the year, whereas the off-season was represented by the remaining 240 days of the year. We describe below each step of the model according to their chronology.

**Initial conditions.** For each simulation, the pathogen population is initially composed of the specialist of the susceptible cultivar (*p*=1). Epidemics are initiated by assuming that plants in susceptible fields are randomly infected with a probability of 0.01 (infected plants at *t*=0 are at an infectious stage).

**Reproduction and mutations.** Infectious sites produce $r$ =5 or 10 effective spores per day resulting from the real number of produced spores, the proportion of spores effectively landing on new hosts after dispersal, and the leaf frailty. Spores belong to the same genotype as their parental lesion with probability $m_{pp}$. We assume that mutations from genotype $p$ to genotype$p'$ arise with probability $m_{pp'}$. In field $i$ and time $t$, the number of spores of each genotype arising from pathogen genotype $p'$, $M_{i,p'}\left(t\right)$, write:

.

Thus, the total number of spores, $Sp_{i,p}\left(t\right)$, belonging to the pathogen genotype $p$ and produced in field $i$ and at time $t$ is computed as .

Pathogen genotypes are classified according to their gain in infection efficacy on the resistant cultivar. Genotype 1 corresponds to the full specialist of the susceptible cultivar (it cannot infect the resistant cultivar) and the genotype $P$ corresponds to the specialist of the resistant cultivar. The other genotypes are considered as generalists with different degree of specialisation since they can infect both cultivars. The probability that a pathogen propagule is of the same genotype as its parental individual was set to $m_{pp}=0.996$. We assume that the pathogen population evolves gradually: a new genotype arises from closely related genotypes by mutation with small gains or losses in infection efficacy by setting $m_{p\left(p-1\right)}=m_{p\left(p+1\right)}=0.002$. Exceptions are the pathogen genotypes with the highest infection efficacy on either the crop or the wild host – these mutate towards less specialised genotypes with a probability of 0.004 to keep their overall mutation rate equal to that of other genotypes.

**Spores dispersal.** Spores migrate from field $i$ to field $j$ with probability $\mu_{i,j}$ computed from:

,

where $A{}_{i}$ and $A_{j}$ are the areas of fields $i$ and $j$, respectively). is the individual dispersal function with an inverse power law shape:

,

where is the Euclidean distance between locations $z$ and $z'$, $b>0$ is a scale parameter and $a>2$determines the weight of the dispersal tail. $a$ is fixed at 3.4 to have a fat tailed dispersal function. The mean dispersal distance is defined as $\mu_{0}=2b/\left(a-3\right)$ and is varied in 2.5%, 10% and 25% of the landscape length (noting that the simulated landscape is square).

$\left[D_{p}\left(t\right)\right]_{i,j}$, the element in the row $i$ and column $j$ of the dispersal matrix $D_{p}\left(t\right)$, gives the number of spores of genotype $p$ dispersing at time $t$ from patch $i$ to patch $j$:

$\left[D_{p}\left(t\right)\right]_{i,\bullet}\text{\textasciitilde Multi}\left(Sp_{i,p}\left(t\right),\left[\mu_{i,1},\mu_{i,2},\cdots\right]\right)$.

Thus, the total numbers of spores of genotype $p$ ($Sp_{j,p}^{tot}\left(t\right)$) arriving in patch $j$ at time $t$ are computed as $Sp_{j,p}^{tot}\left(t\right)=\sum_{i} \left[D_{p}\left(t\right)\right]_{i,j}$.

**Invasion of healthy sites.** Spores arriving on a field contaminate a healthy site with probability $\pi\left(x_{i}\left(t\right)\right)$, where $\pi\left(\cdot\right)$ is an increasing function of $x_{i}\left(t\right)$, the proportion of healthy sites in field $i$ at time $t$. The number of new possible infections (spores that enter in contact with a healthy site), $NewCont_{i}\left(t\right)$, in field $i$ and time $t$ is first computed regardless the pathogen genotype as:

$NewCont_{i}\left(t\right)\sim\text{Bin}\left(S_{i}\left(t-1\right),\pi\left(x_{i}\left(t-1\right)\right)\right)$.

Then, the new possible infections are dispatched among the pathogen genotypes according to their proportion in the set of spores arriving in the field $i$ and time $t$ and following a multinomial distribution:

.

Note that the actual number of spores arriving on a field is taken as a maximum for new possible infections.

**Infection of contaminated sites.** A healthy site receiving a spore (contaminated site) become infected with a probability $e_{p,v\left(i\right)}$, the infection efficacy of pathogen genotype $p$ on the crop cultivar $v\left(i\right)$ cultivated in field $i$. We thus have:

$NewE_{i,p}\left(t\right)\text{\textasciitilde Bin}\left(NewContP_{i,p}\left(t\right),e_{p,v\left(i\right)}\right)$.

We assume a trade-off in infection efficacy on the two crop cultivars (respectively *e_p,RC_* and *e_p,SC_* for the resistant and the susceptible cultivars):: a gain in infection efficacy on the resistant cultivar has a cost in terms of reduced infection efficacy on the susceptible cultivar (and vice-versa). ). Gain and cost are linked through the relationship:

$e_{p,RC}=e_{\max}-1+\left(1-e_{p,SC}^{\frac{1}{\beta}}\right)^{\beta}$ (Eqn1)

with *e_max_*= 0.4 the infection efficacy of a fully specialist, and $\beta$ the global shape of the trade-off curve: the curve is concave when $\beta$ is below unity, linear when $\beta=1$ and convex otherwise. We will refer hereafter to concave curves as weak trade-offs, because they correspond to cases where the cost of being a generalist is low. Similarly, convex curves will be called strong trade-offs. In the simulation experiment, we fixed the maximal infection efficiency at 0.4. The infection efficacies of the other pathogen genotypes were computed from Eqn1, by varying the gain in infection efficacy between 0% and 100% and by considering three values for the trade-off shape, $\beta=0.8$, $\beta=1$ and $\beta=1.2$.

**Transition from latent (E) to infectious (I) sites.** Once infected, the invaded sites remain latent for an average of $\tau=5$ days before becoming infectious. The transition from latent to infectious sites is given by:

$\left\{\begin{matrix} NewI_{i,p}\left(t\right)\text{\textasciitilde Bin}\left(E_{i,p}\left(t-1\right),1-\exp\left(-\frac{1}{\tau}\right)\right) \\ E_{i,p}\left(t\right)=E_{i,p}\left(t-1\right)+NewE_{i,p}\left(t\right)-NewI_{i,p}\left(t\right) \end{matrix}\right.$.

**Removal of infectious sites.** After an average of $T=10$ days of sporulation (infectious period), infectious sites are removed. The transition from infectious sites to removed sites is given by:

$\left\{\begin{matrix} NewR_{i,p}\left(t\right)\text{\textasciitilde Bin}\left(I_{i,p}\left(t-1\right),1-\exp\left(-\frac{1}{T}\right)\right) \\ I_{i,p}\left(t\right)=I_{i,p}\left(t-1\right)+NewI_{i,p}\left(t\right)-NewR_{i,p}\left(t\right) \end{matrix}\right.$.

**Host growth and removal of infected sites.** The plant cover of the crop initiating the cropping season was set to 10% of the field acreage. The crop then grows locally until it reaches the carrying capacity of the field, $K_{i}$, where $K_{i}$ is assumed to be proportional to the area of field $i$. In the following, all the results are expressed as ratios so that they are independent of the constant of proportionality (excepted if very low carrying capacities are considered). In addition we consider that only healthy tissues ($S_{i}$) participate to biomass production (equivalent to a castrating pathogen):

$\left\{\begin{matrix} NewS_{i}\left(t\right)\text{\textasciitilde Bin}\left(S_{i}\left(t-1\right),\delta\left(1-\left(S_{i}\left(t-1\right)+\sum_{p} E_{i,p}\left(t-1\right)+\sum_{p} I_{i,p}\left(t-1\right)\right)/K_{i}\right)\right) \\ S_{i}\left(t\right)=S_{i}\left(t-1\right)+NewS_{i}\left(t\right)-\sum_{p} NewE_{i,p}\left(t\right) \end{matrix}\right.$,

where $\delta=0.1$ is the rate of biomass production when there is no space limitations. At the end of the cropping season 99.9% of the plants are removed randomly, regardless their infection state and $\delta$ was fixed to 0 to keep the total number of sites constant during the off-season.
